# Understanding higher substance use among early adolescent females: A decomposition analysis of adverse childhood experiences, bullying victimization, and depressive symptoms

**DOI:** 10.1016/j.abrep.2026.100744

**Published:** 2026-08-26

**Authors:** Meghan Powers, Stephanie M. Koning, Wei Yang, Minggen Lu, Jennifer McClendon, Kristen Clements-Nolle

**Affiliations:** a Department of Biostatistics, Epidemiology, and Environmental Health, School of Public Health, University of Nevada, Reno, 1664 N. Virginia Street, Reno, NV 89557, USA; b Department of Health Behavior, Policy, and Administration Sciences, School of Public Health, University of Nevada, Reno, 1664 N. Virginia Street, Reno, NV 89557, USA; c Department of Human Development, Family Science, and Counseling, College of Education & Human Development, University of Nevada, Reno, 1664 N. Virginia Street, Reno, NV 89557, USA

**Keywords:** Sex disparity, Substance use, Adolescent, Adverse childhood experiences, Bullying, Depressive symptoms

## Abstract

**Background:**

Historically, U.S. adolescent boys have reported higher substance use than girls, but this has changed in recent years with girls reporting more use. The reasons for this demographic shift are unclear, but girls may be more likely to turn to substance use as a way to cope with stress and internalizing problems.

**Methods:**

We used cross-sectional data from the 2023 Nevada Middle School Youth Risk Behavior Survey (YRBS) (grades 6–8;*n* = 6734). We estimated the adjusted prevalence ratios of girls versus boys past 30-day substance use (electronic vapor products (EVP), alcohol, marijuana, and non-medical prescription pain medicine (NMPPM)) and intervention-relevant factors (adverse childhood experiences (ACEs), bullying victimization, and depressive symptoms). To estimate how much of the potential substance use sex disparities were statistically accounted for by differences in the prevalence of risk factors, we conducted decomposition analyses.

**Results:**

Substance use was higher for girls versus boys: EVP (aPR:2.05, 95%CI:1.62,2.61), alcohol (aPR:1.76, 95%CI:1.39,2.21), marijuana (aPR:1.93, 95%CI:1.43,2.59), and NMPPM (aPR:1.91, 95%CI:1.44,2.54). Girls also reported higher prevalence of intervention-relevant factors. The factors that made the largest contributions to statistically accounting for the substance use sex disparities were depressive symptoms (range:44.9–64.0%), household substance abuse (range:31.0–44.7%), emotional abuse (range:28.4–43.6%), and household mental illness (range:26.9–41.5%).

**Conclusions:**

Recent substance use was approximately twice as high among girls compared to boys. These sex disparities may be statistically accounted for by the higher prevalence of ACEs, electronic bullying, and depressive symptoms among girls. There is a need for sex-specific substance use prevention that is trauma-informed and includes healthy coping strategies.

## Highlights

• Recent substance use was higher among middle school girls than boys.
• Girls also reported higher prevalence of stressors and depressive symptoms.
• Stressors and depression may help explain elevated substance use among girls.

## 1 Introduction

Adolescence is a period of rapid physical, cognitive, and socio-emotional changes (Romer, 2010). It is also a time when youth may begin to experiment with substance use. Adolescent substance use may impair memory, attention, and reasoning ability and increases the risk of motor vehicle crashes, impulsivity, violence perpetration and victimization, emergency department admissions, and substance use disorders (SUD) (Garofoli, 2020; Thomasius et al., 2022). Most adults with SUD initiated substance use during adolescence (Conway et al., 2016; Nelson et al., 2022), and the risk of SUD is greatest among those who start using substances use at an early age (Garofoli, 2020; Nelson et al., 2022).

Historically, adolescent boys in the United States have reported higher substance use than girls (Miech et al., 2025). However, sex differences have narrowed over time, and the most recent national data show that girls report higher prevalences of the substances most commonly used by adolescents: electronic vapor products, alcohol, marijuana and non-medical prescription pain medicine (Centers for Disease Control and Prevention, 2024a; Miech et al., 2025). There is limited information on substance use during early adolescence (10–14 years), but data from the Monitoring the Future Survey show that among 8th graders the prevalence of substance use is much higher among girls and this sex difference is less pronounced when youth reach 12th grade (Miech et al., 2025).

It is not clear what has contributed to the changes in sex differences in early adolescent substance use over time, but gendered mechanisms such as differences in substance use motivations, stress exposures, and internalizing symptoms may offer insight into possible explanatory pathways. Past research has shown that adolescent girls are more likely to turn to substance use as a form of self-medication or as a way to cope with stress and internalizing problems (Agoglia et al., 2020; Dir et al., 2017; Romano et al., 2021). Whereas, boys are more likely to report that their main motivations for substance use are curiosity and sensation seeking (Agoglia et al., 2020). Following the onset of the COVID-19 pandemic, there was an increase in adolescents experiencing childhood adversity (Hertz et al., 2023), bullying victimization (Patte et al., 2024), and depression (Wang et al., 2022). There is a possibility that if girls are more likely to use substances to self-medicate or cope, exposure to stressors and depressive symptoms may help explain recent sex differences adolescent substance use.

Chronic exposure to adverse childhood experiences (ACEs)- such as abuse, neglect, or household challenges - is more commonly reported among adolescent girls (Mersky et al., 2021; Swedo, 2024) and the influence of ACEs on adolescent substance use appears to be stronger among girls than boys (Donovan et al., 2025; Zaidi et al., 2022). While there is conflicting evidence as to whether adolescent boys (Da et al., 2023; Forsberg & Thorvaldsen, 2022; Tharp-Taylor et al., 2009) or girls (Centers for Disease Control and Prevention, 2024a; Kim et al., 2019; Williams et al., 2020) are more likely to be victims of bullying, school-based bullying (Tharp-Taylor et al., 2009) and cyberbullying (Kim et al., 2019) victimization are associated with increased substance use among adolescent girls, but not among boys. Additionally, adolescent girls are more likely to experience depressive symptoms and depression (Dil et al., 2024; Dir et al., 2017; Hernandez et al., 2016; Yoon et al., 2022), and the associations between depressive symptoms and alcohol (Hernandez et al., 2016; Johannessen et al., 2017) and marijuana use (Hernandez et al., 2016) are stronger among adolescent girls than boys.

While adolescent girls generally report higher prevalence of stressors and depressive symptoms, and the influence of these risk factors on substance use is stronger among girls, it is not clear how much of sex disparities in adolescent substance use are statistically accounted for by these intervention-relevant factors. To address this gap, this study will: 1) estimate sex differences in current electronic vapor product, alcohol, marijuana, and non-medical prescription pain medicine use in an early adolescent (10–14 years) population-based sample; and 2) estimate how much of the sex disparities in substance use are partially explained by differences in household ACE exposure, bullying victimization, and depressive symptoms through decomposition analyses. A decomposition framework is useful for determining how much of an observed disparity in an outcome is statistically accounted for by differing distributions of risk factors between two groups (Sudharsanan & Bijlsma, 2021).

## 2 Methods

### 2.1 Participants and procedures

The Youth Risk Behavior Survey (YRBS) is a biennial, surveillance system developed by the Centers for Disease Control and Prevention (CDC) to monitor health behaviors of high school students. However, a limited number of states conduct a middle school YRBS (grades 6–8) (Centers for Disease Control and Prevention, 2024b). To ensure a representative sample of students in 6th–8th grade across all public, charter, and alternative middle schools in Nevada, a two-stage cluster sampling design was used. At the first stage, the 17 school districts in the state were grouped into eight geographic strata, and at the second stage, second period or required English classes were randomly sampled from all participating schools. Passive parental consent was obtained, and student participation was voluntary. Institutional Review Board (IRB) approval was obtained from the University of Nevada, Reno and local school districts as required. Data collection occurred from January to June of 2023.

The student response rate was 76.7%, the school response rate was 98.4%, with an overall response rate of 75.5%. Youth who did not report their sex were excluded from these analyses (*n* = 81), resulting in a final analytic sample of 6734. The mean age of the final analytic sample was 12.61 years old (SD = 0.97).

### 2.2 Measures

#### 2.2.1 Sex

Youth self-reported their sex as female or male.

#### 2.2.2 Intervention-relevant factors

##### 2.2.2.1 Household adverse childhood experiences (ACEs)

Youth were asked about seven different household ACEs: physical abuse, emotional abuse, sexual abuse, witnessing intimate partner violence, household mental illness, household substance abuse, and parent incarceration. All of the household ACE questions are part of CDC's Division of Violence Protection ACE module (DVP, 2021), except for sexual abuse which is a standard question on the high school YRBS. See Supplemental Table 1 for more information on household ACE variables. Youth were coded as affirmative for each individual ACE if they reported ever experiencing that ACE, with the exception of emotional abuse, where youth were coded as affirmative for that ACE if they sometimes, most of the time, or always experienced emotional abuse (DVP, 2021).

##### 2.2.2.2 Bullying victimization

Bullying was defined as “when 1 or more students tease, threaten, spread rumors about, hit, shove, or hurt another student over and over again. It is not bullying when 2 students of about the same strength or power argue or fight or tease each other in a friendly way.” Youth were coded as affirmative for bullying victimization on school property, if they had answered affirmatively to whether they had been bullied on school property during the past 12 months. Youth were coded as affirmative for electronic bullying victimization, if they answered affirmatively to whether they had been electronically bullied during the past 12 months. Both of the bullying victimization questions are standard questions on the high school and middle school YRBS.

##### 2.2.2.3 Depressive symptoms

Youth were asked if they had felt so sad or hopeless almost every day for two weeks or more in a row that they stopped doing some usual activities during the past 12 months. The depressive symptoms question is a standard question on the high school YRBS.

#### 2.2.3 Substance use

Youth were asked how many days/times they had used electronic vapor products (EVP), alcohol, marijuana, and non-medical prescription pain medicine (NMPPM) in the past 30-days. NMPPM was defined as “the use of prescription pain medicine without a doctor's prescription or differently than how a doctor told you to use it,” and youth were provided codeine, Vicodin, OxyContin, Hydrocodone, and Percocet as examples of NMPPM. The EVP, alcohol, and marijuana questions are standard questions on the high school YRBS and the NMPPM question is an optional YRBS question. Youth who reported they had used that substance on at least one day or at least one time were coded as affirmative for past 30-day use of that substance.

#### 2.2.4 Sociodemographics

Youth self-reported their age and race/ethnicity (Hispanic, Non-Hispanic White, Non-Hispanic Black, Non-Hispanic Multiple/Other Race). Title I eligibility of each participating school was obtained from the State Department of Education.

### 2.3 Statistical analysis

The data were weighted at the state and regional levels for sex, grade, and race/ethnicity. Multiple imputation was performed using chained equations through the fully conditional specification method (FCS) (Liu & De, 2015) for the intervention-relevant factors (range of 3.3% for electronic bullying victimization to 7.4% for parent incarceration), past 30-day substance use (range of 6.1% for alcohol to 12.0% for NMPPM), age (0.3%), and race/ethnicity (0.4%). Boys had a significantly higher prevalence of missing data compared to girls for the intervention-relevant factors, alcohol, and marijuana. There was no missing data for sex and Title I eligibility status. Twenty imputations were performed, and estimates were pooled across imputed datasets using Rubin's rules.

We estimated the weighted percentages and confidence intervals for sociodemographics, substance use, household ACEs, bullying victimization, and depressive symptoms for the analytic sample. Next, we ran weighted modified Poisson regression with a log link function to estimate the unadjusted and adjusted prevalence ratios of girls' versus boys' substance use, household ACEs, bullying victimization, and depressive symptoms controlling for sociodemographics (age, race/ethnicity, and Title I eligibility status). Variance was estimated with the robust sandwich estimator.

To estimate how much of the potential substance use sex disparities were statistically accounted for by differences in the prevalence of intervention-relevant factors, we ran decomposition analyses using R package *cfdecomp*. For the decomposition analyses, the weighted adjusted prevalence ratios of girls' versus boys' substance use were used as the natural-course prevalence ratios, and the *cfdecomp* R package was used to run a counterfactual decomposition using the parametric g-formula and Monte Carlo (MC) integration (Sudharsanan & Bijlsma, 2021). Within each of the 20 imputed datasets, we ran 250 bootstrap replicates, which accounted for the stratification and clustering of the sample, and ran 50 MC integrations per bootstrap replicate. The bootstrap resampling occurred at the cluster level. The counterfactual decomposition represents what the sex disparity in substance use would be if girls had the same prevalence of the intervention-relevant factors as boys (Jackson & VanderWeele, 2018).

The natural-course prevalence ratio reflects the observed girl-versus-boy disparity of each substance given the observed girl-versus-boy distribution of an intervention-relevant factor. The counterfactual prevalence ratio reflects the estimated girl-versus-boy disparity of each substance if girls had the same prevalence of an intervention-relevant factor as boys. The percent contribution reflects the contribution of an intervention-relevant factor to the girl-versus-boy substance use disparity as a percentage reduction, by comparing the natural-course prevalence ratio and the counterfactual prevalence ratio. We ran unique models for each intervention-relevant factor and all substance use outcomes, controlling for sociodemographics. Since each factor was modeled separately, the percent contributions are not mutually exclusive and cannot be summed across factors.

All analyses accounted for the complex survey design of the data and accounted for the strata and cluster sampling; SAS 9.4 (SAS Institute, Cary, NC) was used for the descriptive analyses and estimated adjusted prevalence ratios, and R Statistical Software (v4.2.3, R Core Team, 2023) was used for the decomposition analyses.

## 3 Results

### 3.1 Sample characteristics

Table 1 presents the descriptive characteristics for the full sample. Nearly half of youth were female (48.5%), 65.1% of youth were either 12 or 13 years old, 45.7% were Hispanic, and 76.3% attended a Title I eligible school. Past 30-day substance use among middle school students included EVP (9.5%), alcohol (8.2%), marijuana (6.7%), and NMPPM (6.7%). The most commonly reported household ACEs were emotional abuse (36.0%), household mental illness (29.6%), household substance abuse (22.1%), and witnessing intimate partner violence (17.7%). Almost a quarter of the sample reported being bullied on school property (23.8%), 16.4% reported being a victim of electronic bullying, and 34.7% reported depressive symptoms.

**Table 1:** Weighted sample characteristics (2023 nevada middle school youth risk behavior survey, n = 6734).

Sample characteristics	%^1^ (95%CI)
Sociodemographics	
Sex	
Female	48.5 (45.6, 51.4)
Male	51.5 (48.6, 54.4)
Age	
11 years or younger	14.2 (11.0, 17.4)
12 years	32.8 (29.3, 36.3)
13 years	32.3 (29.2, 35.3)
14 years or older	20.7 (17.1, 24.3)
Race/Ethnicity	
Hispanic	45.7 (42.1, 49.3)
Non-Hispanic White	27.8 (24.7, 30.9)
Non-Hispanic Black	12.1 (10.6, 13.6)
Non-Hispanic Multiple/Other Race	14.3 (12.5, 16.2)
Title I Eligible School	76.3 (69.7, 82.9)
Past 30-Day Substance Use	
Electronic Vapor Product	9.5 (8.2, 10.7)
Alcohol	8.2 (7.2, 9.3)
Marijuana	6.7 (5.6, 7.8)
Non-Medical Prescription Pain Medicine	6.7 (5.9, 7.5)
Stressors and Depressive Symptoms	
Physical Abuse	14.7 (13.5, 16.0)
Emotional Abuse	36.0 (34.0, 38.0)
Sexual Abuse	6.2 (5.3, 7.2)
Witness Intimate Partner Violence	17.7 (16.1, 19.2)
Household Mental Illness	29.6 (27.9, 31.3)
Household Substance Abuse	22.1 (20.5, 23.6)
Parent Incarceration	16.4 (14.9, 17.9)
Bullying Victimization on School Property	23.8 (22.3, 25.3)
Electronic Bullying Victimization	16.4 (15.0, 17.8)
Depressive Symptoms	34.7 (32.7, 36.7)

CI = confidence interval

1 Weighted, column percent.

### 3.2 Sex differences in early adolescent substance use, household ACEs, bullying victimization, and depressive symptoms

After adjusting for age, race, and Title 1 eligibility status, girls had a higher prevalence of past 30-day substance use compared to boys: EVP (aPR: 2.05, 95%CI: 1.62, 2.61), alcohol (aPR: 1.76, 95%CI: 1.39, 2.21), marijuana (aPR: 1.93, 95%CI: 1.43, 2.59), and NMPPM (aPR: 1.91, 95%CI: 1.44, 2.54) (Table 2).

**Table 2:** Sex differences in the prevalence of substance use, stressors, and depressive symptoms (2023 Nevada Middle School Youth Risk Behavior Survey, n = 6734).

Variable	PR (95%CI) ref. = boys	aPR^1^ (95%CI) ref. = boys
Past 30-Day Substance Use		
Electronic Vapor Product	2.06 (1.62, 2.63)	2.05 (1.62, 2.61)
Alcohol	1.74 (1.38, 2.20)	1.76 (1.39, 2.21)
Marijuana	1.91 (1.41, 2.59)	1.93 (1.43, 2.59)
Non-Medical Prescription Pain Medicine	1.92 (1.44, 2.55)	1.91 (1.44, 2.54)
Stressors and Depressive Symptoms		
Physical Abuse	1.44 (1.22, 1.69)	1.44 (1.22, 1.69)
Emotional Abuse	1.56 (1.42, 1.71)	1.56 (1.42, 1.71)
Sexual Abuse	2.53 (1.89, 3.38)	2.53 (1.89, 3.37)
Witness Intimate Partner Violence	1.61 (1.41, 1.83)	1.60 (1.39, 1.82)
Household Mental Illness	1.66 (1.50, 1.85)	1.67 (1.51, 1.85)
Household Substance Abuse	1.83 (1.63, 2.05)	1.84 (1.63, 2.07)
Parent Incarceration	1.31 (1.13, 1.51)	1.31 (1.14, 1.52)
Bullying Victimization on School Property	1.29 (1.14, 1.46)	1.29 (1.14, 1.45)
Electronic Bullying Victimization	1.72 (1.46, 2.01)	1.71 (1.46, 2.01)
Depressive Symptoms	1.92 (1.74, 2.13)	1.92 (1.74, 2.12)

PR = prevalence ratio; CI = confidence interval; aPR = adjusted prevalence ratio

1 aPR adjusts for age, race/ethnicity, and Title I eligibility status; aPR referent group is boys.

Middle school girls also had significantly higher prevalence of all stressors and depressive symptoms compared to boys: physical abuse (aPR: 1.44, 95%CI: 1.22, 1.69), emotional abuse (aPR: 1.56, 95%CI: 1.42, 1.71), sexual abuse (aPR: 2.53, 95%CI: 1.89, 3.38), witnessing intimate partner violence (aPR: 1.61, 95%CI: 1.41, 1.83), household mental illness (aPR: 1.66, 95%CI: 1.50, 1.85), household substance abuse (aPR: 1.83, 95%CI: 1.63, 2.05), parent incarceration (aPR: 1.31, 95%CI: 1.13, 1.51), bullying victimization on school property (aPR: 1.29, 95%CI: 1.14, 1.45), electronic bullying victimization (aPR: 1.71, 95%CI: 1.46, 2.01), and depressive symptoms (aPR: 1.92, 95%CI: 1.74, 2.12).

### 3.3 Decomposition analyses

Table 3 presents the decomposition analyses. The counterfactual aPR represents what the sex disparity in early adolescent substance use would be if girls had the same prevalence of the intervention-relevant factors as boys, and the percent contribution represents how much of the overserved sex disparity is helped explained by the differing distribution of that factor between girls and boys.

**Table 3:** Estimates of the contribution of intervention-relevant factors for sex disparities in past 30-day substance use using the counterfactual decomposition method (2023 nevada middle school youth risk behavior survey, n = 6734).

Intervention-relevant factors	Electronic vapor products			Alcohol			Marijuana			Non-medical prescription pain medicine		
	Natural Course^1^	Counterfactual^2^	Percent Contribution^3^	Natural Course^1^	Counterfactual^2^	Percent Contribution^3^	Natural Course^1^	Counterfactual^2^	Percent Contribution^3^	Natural Course^1^	Counterfactual^2^	Percent Contribution^3^
	aPR^4^ (95%CI) ref. = boys	aPR^4^ (95%CI) ref. = boys	% (95%CI)	aPR^4^ (95%CI) ref. = boys	aPR^4^ (95%CI) ref. = boys	% (95%CI)	aPR^4^ (95%CI) ref. = boys	aPR^4^ (95%CI) ref. = boys	% (95%CI)	aPR^4^ (95%CI) ref. = boys	aPR^4^ (95%CI) ref. = boys	% (95%CI)
Physical Abuse	2.00 (1.65, 2.35)	1.87 (1.54, 2.20)	13.1 (4.2, 22.0)	1.57 (1.30, 1.85)	1.46 (1.21, 1.71)	19.9 (6.0, 33.9)	1.75 (1.35, 2.15)	1.61 (1.25, 1.97)	18.8 (5.3, 32.3)	1.76 (1.41, 2.11)	1.59 (1.28, 1.91)	22.6 (8.3, 36.9)
Emotional Abuse	1.99 (1.63, 2.34)	1.71 (1.40, 2.02)	28.4 (18.0, 38.8)	1.56 (1.30, 1.83)	1.35 (1.12, 1.58)	39.4 (20.8, 58.0)	1.74 (1.35, 2.13)	1.48 (1.15, 1.81)	36.1 (19.1, 53.1)	1.75 (1.40, 2.11)	1.43 (1.14, 1.73)	43.6 (25.8, 61.3)
Sexual Abuse	2.01 (1.66, 2.36)	1.82 (1.50, 2.14)	19.5 (9.7, 29.3)	1.58 (1.31, 1.85)	1.45 (1.20, 1.70)	22.7 (7.7, 37.8)	1.77 (1.36, 2.18)	1.58 (1.21, 1.95)	25.0 (8.9, 41.1)	1.77 (1.41, 2.12)	1.60 (1.27, 1.94)	21.7 (6.7, 36.7)
Witness Intimate Partner Violence	2.02 (1.66, 2.38)	1.82 (1.50, 2.14)	19.6 (10.4, 28.7)	1.58 (1.31, 1.84)	1.42 (1.18, 1.65)	28.3 (13.3, 43.4)	1.76 (1.36, 2.17)	1.55 (1.20, 1.90)	29.5 (14.1, 44.8)	1.77 (1.41, 2.13)	1.58 (1.25, 1.90)	25.5 (11.2, 39.9)
Household Mental Illness	1.99 (1.64, 2.34)	1.73 (1.42, 2.03)	26.9 (16.3, 37.5)	1.57 (1.29, 1.84)	1.37 (1.12, 1.61)	36.3 (17.6, 55.0)	1.74 (1.34, 2.13)	1.44 (1.12, 1.76)	41.5 (22.3, 60.8)	1.75 (1.39, 2.10)	1.46 (1.16, 1.76)	39.6 (22.6, 56.7)
Household Substance Abuse	1.99 (1.63, 2.34)	1.68 (1.39, 1.98)	31.0 (20.4, 41.7)	1.56 (1.29, 1.84)	1.36 (1.12, 1.59)	37.9 (20.2, 55.6)	1.73 (1.34, 2.11)	1.41 (1.10, 1.72)	44.7 (25.3, 64.1)	1.75 (1.39, 2.10)	1.52 (1.21, 1.83)	31.0 (15.4, 46.5)
Parent Incarceration	2.02 (1.66, 2.38)	1.93 (1.60, 2.27)	8.5 (−0.1, 17.2)	1.58 (1.30, 1.85)	1.53 (1.27, 1.78)	8.6 (−3.4, 20.6)	1.77 (1.37, 2.17)	1.68 (1.31, 2.05)	11.6 (−0.9, 24.1)	1.77 (1.41, 2.13)	1.70 (1.36, 2.04)	9.0 (−3.1, 21.0)
Bullying Victimization on School Property	2.00 (1.64, 2.35)	1.93 (1.59, 2.27)	6.9 (−1.5, 15.2)	1.57 (1.29, 1.84)	1.51 (1.25, 1.78)	9.1 (−3.3, 21.6)	1.75 (1.35, 2.15)	1.69 (1.30, 2.08)	7.9 (−4.2, 20.0)	1.75 (1.39, 2.10)	1.67 (1.33, 2.01)	10.5 (−2.0, 23.1)
Electronic Bullying Victimization	2.00 (1.65, 2.36)	1.80 (1.48, 2.11)	20.9 (11.4, 30.4)	1.57 (1.30, 1.85)	1.42 (1.17, 1.67)	27.2 (11.3, 43.1)	1.75 (1.35, 2.15)	1.56 (1.20, 1.92)	25.9 (10.4, 41.5)	1.76 (1.41, 2.11)	1.58 (1.26, 1.90)	23.7 (9.4, 37.9)
Depressive Symptoms	1.99 (1.63, 2.35)	1.55 (1.27, 1.83)	44.9 (32.5, 57.4)	1.56 (1.29, 1.84)	1.25 (1.02, 1.48)	57.4 (31.6, 83.3)	1.74 (1.34, 2.14)	1.34 (1.03, 1.65)	55.7 (32.2, 79.2)	1.75 (1.40, 2.10)	1.28 (1.02, 1.55)	64.0 (41.2, 86.9)

aPR = adjusted prevalence ratio; CI = confidence interval

1 The natural-course prevalence ratio reflects the observed girl-versus-boy disparity of each substance given the observed girl-versus-boy distribution of an intervention-relevant factor.

2 The counterfactual prevalence ratio reflects the estimated girl-versus-boy disparity of each substance if girls had the same prevalence of an intervention-relevant factor as boys.

3 The percent contribution reflects the contribution of an intervention-relevant factor to the girl-versus-boys substance use disparity as a percentage reduction, by comparing the natural-course prevalence ratio and the counterfactual prevalence ratio. Since each factor was modeled separately, the percent contributions are not mutually exclusive and cannot be summed across factors.

4 aPR adjusts for age, race/ethnicity, and Title I eligibility status; aPR referent group is boys.

The intervention-relevant factors that made the largest contributions to helping explain the disparity in past 30-day substance use between girls and boys were depressive symptoms (range of 44.9% for EVP to 64.0% for NMPPM), household substance abuse (range of 31.0% for EVP and NMPPM to 44.7% for marijuana), emotional abuse (range of 28.4% for EVP to 43.6% for NMPPM), and household mental illness (range of 26.9% for EVP to 41.5% for marijuana).

Parent incarceration and bullying victimization on school property did not make significant independent contributions toward explaining the sex disparity in early adolescent substance use.

## 4 Discussion

In this representative sample of middle school students (6th–8th grade), girls had about twice the prevalence of past 30-day EVP, alcohol, marijuana, and NMPPM use compared to boys. Our finding that girls reported more substance use than boys are consistent with literature among older adolescents (Centers for Disease Control and Prevention, 2024a; Miech et al., 2025). There is limited evidence on the sex differences among early adolescents, but our findings do align with national data among 8th graders (Miech et al., 2025). Our findings build upon this limited set of evidence by including a broader range of early adolescents (6th–8th graders) and including underreported substances, like NMPPM. Together, these findings highlight the importance of early intervention, as youth who initiate substance use at an early age have an increased risk of SUD (Garofoli, 2020; Nelson et al., 2022). The early adolescent developmental period is critical for substance use prevention efforts, and understanding the preventable and intervention-relevant factors that contribute to sex differences in substance use is critical.

Our decomposition framework used counterfactual simulation and allowed us to estimate what the sex disparity in substance use among middle school students would be if girls had the same prevalence of stressors and depressive symptoms as boys. We found that depressive symptoms statistically accounted for 44.9%–64.0% of the higher prevalence of substance use among middle school girls. Following depressive symptoms, household ACEs - such as emotional abuse, household mental illness, and household substance abuse – had the next strongest contribution (26.9%–44.7%). Our findings support the necessity of multilevel primary and secondary prevention efforts addressing these stressors.

There are multiple strategies for primary and secondary prevention of ACEs and electronic bullying. At the family level, parenting/co-parenting classes and home visiting programs have been shown to be promising at preventing ACEs (Kinsey et al., 2024). At the school level, school connectedness may act as a form of secondary prevention against stressors for both high school (McCabe et al., 2026) and middle school students (Clements-Nolle et al., 2022). ACE prevention may also occur at the community level, by leveraging community resources, such as increasing access to transportation, to mitigate the effects of lower incomes on ACEs (Blair et al., 2019). At the policy level, ACEs may be prevented through increased economic support, such as family-friendly work policies and tax credits (Ottley et al., 2022).

Additionally, while we did not explore substance use motivations, our findings align with the theory that the higher substance use among early adolescent girls is influenced by their motivation to use substances as a form of coping (Agoglia et al., 2020; Dir et al., 2017; Romano et al., 2021). Learning healthy coping behaviors is important for early adolescent girls, as they experience stress differently than boys in part due to neurobiological differences in stress responses. During adolescence, girls have a heightened stress reactivity compared to boys (Dir et al., 2017). So, early adolescent girls may especially benefit from assistance with finding healthy ways to deal with chronic stressors, instead of turning to self-medication with substance use. There is a critical need for substance use interventions that include a focus on addressing depressive symptoms and trauma among middle school girls. School-based interventions that utilize cognitive behavioral therapy (CBT) programs have been found to reduce depression among secondary-school populations (Zhang et al., 2023), and trauma-focused CBT interventions are beneficial to early adolescent girls (Auslander et al., 2017). These CBT programs may be especially beneficial for girls as they often include teaching healthy and adaptive coping skills (Dir et al., 2017), which may replace unhealthy coping behaviors, such as substance use.

We found it noteworthy that cyberbullying victimization was a significant contributor to understanding the higher prevalence of substance use among middle school girls, but bullying victimization on school property was not. Our findings align with prior research, which found that adolescent cyberbullying victimization has stronger associations with substance use (Rostam-Abadi et al., 2024) and emotional problems (Kim et al., 2018) than bullying victimization on school property does. The contribution of cyberbullying to the sex disparity in early adolescent substance use is concerning, given that half of teens have a daily screen time of four or more hours (Zablotsky et al., 2024).

A key strength of this paper is our focus on early adolescence (grades 6–8), a demographic that remains understudied despite evidence indicating that early initiation of substance use increases the risk of SUD (Garofoli, 2020; Nelson et al., 2022). Our sample of early adolescents is a large, representative sample of middle school students across a state, with 98.4% of middle schools participating. Our sample was also racially/ethnic diverse, with nearly half of adolescents reporting themselves as Hispanic. Intersectional analyses were beyond the scope of the current study, but should be explored in future research. Another strength of this study is our methodology; we used decomposition analysis to quantify how much of the sex disparity is statistically accounted for by intervention-relevant factors. Finally, we examined multiple substances, including less researched substances such as NMPPM.

However, this study is not without limitations. First, we analyzed cross-sectional data, so we cannot establish a temporal relationship between the intervention-relevant factors and past 30-day substance use and there is a possibility of reverse or bidirectional associations. Second, there are additional risk factors – such as social media use, relational stress, help-seeking behaviors, or anxiety – that may also play an important part in understanding why early adolescent girls have higher substance use than boys. Third, it is likely that many of the intervention-relevant factors we examined are interrelated and there may be mediated effects, but it was beyond the scope of this paper to examine multiple factors simultaneously. The percent contribution of each factor should be interpreted independent of other factors. Fourth, single items were used to measure depressive symptoms, which may affect the interpretation of the intervention-relevant factors. Future research should use validated, multi-item measures. Fifth, girls are more likely to self-report stressors (O'Gorman et al., 2024) and depressive symptoms (Shi et al., 2021) than boys, so if girls were also more likely to self-report substance use, there may be a bias away from the null. Sixth, boys had a higher prevalence of missing data than girls, as such, the imputed values may not fully capture the true distribution of these variables among boys. Seventh, our findings come from only the state of Nevada, which has a high population of Hispanic early adolescents. As such, our findings may not be generalizable to other states or countries. Finally, while we accounted for sociodemographics, we cannot rule out residual confounding.

In conclusion, we found that middle school-aged girls had almost twice the prevalence of past 30-day EVP, alcohol, marijuana, and NMPPM use compared to boys. These sex disparities may be statistically accounted for, in part, by a higher prevalence of depressive symptoms and chronic stressors – such as emotional abuse, household mental illness, and household substance abuse- among girls. There is an urgent need for primary and secondary prevention of stressors and for sex-specific substance use prevention efforts that are trauma-informed and includes a focus on healthy coping strategies.

## CRediT authorship contribution statement

**Meghan Powers:** Writing – original draft, Methodology, Investigation, Formal analysis, Conceptualization. **Stephanie M. Koning:** Writing – review & editing, Methodology. **Wei Yang:** Writing – review & editing, Investigation. **Minggen Lu:** Writing – review & editing, Formal analysis. **Jennifer McClendon:** Writing – review & editing. **Kristen Clements-Nolle:** Writing – review & editing, Supervision, Methodology, Investigation, Conceptualization.

## Funding

The research presented in this paper was supported by funding from the 10.13039/100000030Centers for Disease Control and Prevention (CDC) under award DP-24-0139 and supplemental funding from the Nevada Division of Public and Behavioral Health.

## Declaration of competing interest

The authors declare that they have no known competing financial interests or personal relationships that could have appeared to influence the work reported in this paper.

## Data Availability

The authors do not have permission to share data.

## Appendix A Supplementary data

Supplementary material: Supplementary material - 2023 Nevada Middle School Youth Risk Behavior Survey (YRBS) household adverse childhood experiences (ACE) variables.
