# Supplementary material for: Understanding higher substance use among early adolescent females: A decomposition analysis of adverse childhood experiences, bullying victimization, and depressive symptoms
Source: Addict Behav Rep. 2026 Aug 26;24:100744. doi: 10.1016/j.abrep.2026.100744 (PMC13543975; doi:10.1016/j.abrep.2026.100744)
Supplement: Supplementary file 1 [file mmc1.docx]

**Table S1.**

*2023 Nevada Middle School Youth Risk Behavior Survey (YRBS) household adverse childhood experiences (ACE) variables*

| Household ACE | Question Text | Response Coding |
| --- | --- | --- |
| Physical Abuse | Have you ever been hit, beaten, kicked, or physically hurt in any way by an adults? (Do not include being spanked for  bad behavior.) | Affirmative = Yes  Negative = No |
| Emotional Abuse | How often has an adult in your home ever sworn at you, insulted you, or put you down? | Affirmative = Sometimes; Most of the time; Always  Negative = Never; Rarely |
| Sexual Abuse | Have you ever been physically forced to have sexual intercourse when you did not want to? | Affirmative = Yes  Negative = No |
| Witnessing Intimate Partner Violence | Have you ever seen or heard adults in your home slap, hit, kick, punch, or beat each other up? | Affirmative = Yes  Negative = No |
| Household Mental Illness | Have you ever lived with someone who was depressed, mentally ill, or suicidal? | Affirmative = Yes  Negative = No; Not Sure |
| Household Substance Abuse | Have you ever lived with someone who was a problem drinker or alcoholic or abused street or prescription drugs? | Affirmative = Yes  Negative = No; Not Sure |
| Parent Incarceration | Have you ever been separated from a parent or guardian because they went to jail, prison, or a detention center? | Affirmative = Yes  Negative = No; Not Sure |
